# Identification and Functional Analysis of lncRNAs Responsive to Hypoxia in *Eospalax fontanierii*

**DOI:** 10.3390/cimb43030132

**Published:** 2021-11-05

**Authors:** Zhiqiang Hao, Mingfang Han, Juanjuan Guo, Guanglin Li, Jianping He, Jingang Li

**Affiliations:** National Engineering Laboratory for Resource Development of Endangered Crude Drugs in Northwest China, Key Laboratory of the Ministry of Education for Medicinal Resources and Natural Pharmaceutical Chemistry, College of Life Science, Shaanxi Normal University, Xi’an 710119, China; haozhiqiang@snnu.edu.cn (Z.H.); hanym@snnu.edu.cn (M.H.); gbei@snnu.edu.cn (J.G.); glli@snnu.edu.cn (G.L.)

**Keywords:** hypoxia adaptation, *Eospalax fontanierii*, transcriptome, LncRNAs

## Abstract

Subterranean rodents could maintain their normal activities in hypoxic environments underground. *Eospalax fontanierii*, as one kind of subterranean rodent found in China can survive very low oxygen concentration in labs. It has been demonstrated that long non-coding RNAs (lncRNAs) have important roles in gene expression regulations at different levels and some lncRNAs were found as hypoxia-regulated lncRNAs in cancers. We predicted thousands of lncRNAs in the liver and heart tissues by analyzing RNA-Seq data in *Eospalax fontanierii*. Those lncRNAs often have shorter lengths, lower expression levels, and lower GC contents than mRNAs. Majors of lncRNAs have expression peaks in hypoxia conditions. We found 1128 DE-lncRNAs (differential expressed lncRNAs) responding to hypoxia. To search the miRNA regulation network for lncRNAs, we predicted 471 and 92 DE-lncRNAs acting as potential miRNA target and target mimics, respectively. We also predicted the functions of DE-lncRNAs based on the co-expression networks of lncRNA-mRNA. The DE-lncRNAs participated in the functions of biological regulation, signaling, development, oxoacid metabolic process, lipid metabolic/biosynthetic process, and catalytic activity. As the first study of lncRNAs in *Eospalax fontanierii*, our results show that lncRNAs are popular in transcriptome widely and can participate in multiple biological processes in hypoxia responses.

## 1. Introduction

Subterranean rodents suffer from adverse environmental factors underground, such as hypoxia and hypercapnia. To cope with this, they have evolved and adapted in a long-term period. For blind mole rats, they can survive under 3% O_2_ for more than 11 h without obvious damage, while rats survive only 2.5 h under the same condition [1]. Although mice or rats serve as model animals for medical research, they are limited in hypoxia studies as they are sensitive under severe hypoxia. Blind mole rats and naked mole rats attract scientists’ attention for their cancer-resistant, long-lived, and hypoxia-adaptive traits. AS with blind mole rats, *Eospalax fontanierii* (*E. fontanierii*) are strictly subterranean rodents that survive in habitats poor in oxygen and rich in carbon dioxide and ammonia.

The subterranean *E. fontanierii* belongs to the Myospalactinae subfamilies of the family Spalacidae and is mainly distributed in *the Loess Plateau* of China [2,3,4]. *E. fontanierii* is close with *Nannospalax ehrenbergi* and *Rhizomys sinensis* in the evolutionary distance and they all belong to the family Spalacidae [5]. In the lab, *E. fontanierii* can survive more than 10 h under 4% O_2_ without injury, *Sprague Dawley rats* (*SD rats*) survive only 6 h under the same conditions [6]. By analyzing the blood components, increased red blood cells and hemoglobin concentration, and decreased coagulation rate under hypoxia in *E. fontanierii* we can better understand their adaptation to hypoxia [7,8]. Under hypoxia, tissues generate reactive oxygen species to damage the integrity of cell components, and *E. fontanierii* can overcome the oxidative stress by increasing the expression levels or the protein activities of antioxidant genes, such as metallothionein, cystathionine beta-synthase, superoxide dismutase, catalase, and glutathione reductase [9,10]. Energy supply is important for normal activities underground, and enhanced fructose-driven glycolysis is found to accelerate the supply of energy in *E. fontanierii* under hypoxia [11]. However, more aspects of molecular mechanisms for the adaptation to hypoxia in *E. fontanierii* remain unknown.

Long noncoding RNAs (LncRNAs) are a class of RNAs with a lack of coding potential and length of more than 200 bp. LncRNAs play important roles in regulating gene expression in transcriptional and posttranscriptional methods [12,13]. Many studies suggest that LncRNAs involve almost all life processes [14,15,16]. Recent studies show that LncRNAs can be induced by hypoxia-inducible factor 1 and were involved in the regulatory mechanisms of gene expression under hypoxia during the progression of different types of cancers [17,18,19,20]. Besides, research in naked mole-rat showed that LncRNAs may have important effects on anticancer mechanisms [21]. As lncRNAs play potential functions in hypoxic stress, it is necessary to identify and profile lncRNAs responding to hypoxia response in subterranean rodents (for example, *E. fontanierii*).

In this study, we identified for the first time the transcriptome-wide lncRNA in *E. fontanierii* from liver and heart transcriptomes under different oxygen levels. The lncRNAs associated with hypoxia stress were identified and their functions were explored. Our results provide new insight into the hypoxia adaptation in *E. fontanierii*, which broaden our knowledge about the adaptation mechanisms of subterranean rodents.

## 2. Materials & Methods

### 2.1. Sampling and Data Collection

As the species (*E. fontanierii*) is considered an important agricultural pest and is not protected under any local, regional, national, or international decree, we purchase those individuals from local farmers. Eighteen individuals of *E. fontanierii* (male and female, 220–280 g) were captured from agricultural land in Yan’an (N 35°09′, E 109°22′), Shaanxi Province, China. The species conservation status is ‘least concern’ (LC), and the population trend is unknown. All animals were captured and treated humanely according to guidelines of the Care and Uses of Laboratory Animals of China, and all of the procedures were approved by the Animal Care and Use Committee of Shaanxi Normal University (SNNU-IACUC-EAC-008-2010). Field experiments were approved by the Shaanxi Normal University, College of Life Science (project number: 18.11.20). Each animal was housed in a separate cage [475 L × 350 W × 200 H (mm)] maintained at 21 ± 1 °C under dark environment. All animals were allowed free access to food (carrots). The adapted *E. fontanierii* were randomly divided into three groups (*n* = 6 per group): 6.5% O_2_ 6 h (acute hypoxia), 10.5% O_2_ 44 h (chronic hypoxia), and 21% O_2_ (normoxia). In the normoxia group (21% O_2_), animals breathed normal air for one week. In the chronic hypoxia group, animals were placed in a hypoxia chamber containing 10.5% O_2_ for 44 h. In the acute hypoxia group, animals were placed in 6.5% O_2_ hypoxia chamber for 6 h. The chamber was ventilated with nitrogen to maintain a constant oxygen concentration, which was monitored using a JRC-1020 thermo-magnetic analyzer. The animals were anesthetized with an intraperitoneal injection of pentobarbital (45 mg/kg) and sacrificed to collect fresh tissues and frozen into liquid nitrogen immediately. Total RNA was extracted using an RNA Simple Total RNA kit (TaKaRa) according to the instructions. As the transcriptome data of *E. fontanierii* heart and liver tissues treated by different oxygen levels were published by our previous studies, those data were downloaded from NCBI database under BioProject accession number PRJNA497961 [8,10]. Those data are paired-end reads with a length of about 150 bp. The mature sequence of miRNA from four rodential species (*Cricetulus griseus*, *Cavia porcellus*, *Mus musculus*, *Rattus norvegicus*) were downloaded from miRbase (version 22.0) [22].

### 2.2. Assembly and Annotation

The RNA-Seq data were cleaned by removing the primer sequences, adapters, and low-quality bases with fastp (v0.20.1) [23]. Then all data were assembled into one transcript set by Trinity (v2.4) [24]. To annotated the functions of transcript set, blastx were performed for all transcripts against Nr database (E-value < 1e-3). Software Blast2GO Command Line (v1.0.2) (-https://www.biobam.com/blast2go-command-line-tools/, latest accessed data: 2/11/2021) were used to find the GO terms for annotated transcripts with blastx output xml file as input (threshold *E*-value < 1e-6, command: Blast2GO_HOME/blast2go_cli.run -properties cli.prop -loadfasta input.fasta -loadblast blastResult.xml -mapping -annotation -saveb2g -savedat -annex -useobo go-basic.obo) [25]. We considered the transcripts with homologies in Nr database and GO term annotations as mRNAs for next analysis. Website tool KOBAS3.0 was used to find functional annotations of mRNAs in the Kyoto Encyclopedia of Genes and Genomes (KEGG) [26].

### 2.3. Pipeline for lncRNA Identification

To identify the lncRNAs in transcript set, we designed a pipeline by multiple bioinformatics methods. Firstly, the sequence length of transcripts should be more than 200 nt. After this, the longest ORF (open reading frame) in transcripts should be not more than 300 nt from start codon to stop codon. The longest ORF were identified by TransDecoder (command1: TransDecoder. LongOrfs -t input.fasta; command2: TransDecoder.predict—t input.fasta) (v3.0.1) [24]. Subsequently, BLASTx (v2.6.0+) searching for transcripts were performed against the Swiss-Prot (*E*-value < 1e-10) and Nr (*E*-value < 1e-10). The matched transcripts were filtered. And then, we searched the Pfam motifs in peptide sequences of transcript using HMMER (version: 3.1b2) (command: phmmer -E 1e-5 -cpu 8 -pfamtblout output input.pep Pfam-A.hmm) [27,28]. Those transcripts with peptide sequence containing Pfam motifs were removed. CPC (coding potential calculator) and CPAT (threshold:0.44) were used to filter the potential coding transcripts [29,30]. Rfam database were used to remove known noncoding RNAs, such as small nucleolar RNAs (snoRNAs), small nuclear RNAs (snRNAs), transfer RNAs (tRNAs), and ribosomal RNAs (rRNAs) (blastn, *E*-value < 1e-5) [31].

### 2.4. Expression Analysis and DE-lncRNA Identification

We calculated the transcript expression levels by the script from Trinity toolkit (command: TRINITY_HOME/util/align_and_estimate_aubundance. pl —transcript Trinity.fasta –seqType fq –left reads_1.fq –right reads_2.fq –est_method RSEM –aln_method bowtie –trinity_mode –prep_reference –out_dir rsem_outdir). DESeq2 (v1.26.0) of R package was used to identify DE-lncRNAs [32]. Fold change for expression >2 or <0.5 and *p*-value < 0.05 (Wald test) were considered as DE-lncRNAss. The perl script (run_DE_analysis.pl) in Trinity2.4 toolkits was used for the identification of DE-lncRNAs by integrating DESeq2 (command: TRINITY_HOME/Analysis/Differential_Expression/run_DE_analysis.pl –matrix counts.matrix –method DESeq2 –samples_file samples_described.txt) [24].

### 2.5. Real-Time PCR Validation of DE-lncRNAs

Three DE-lncRNAs predicted in response to hypoxia in liver or heart were selected to validate the reliability of the DE-lncRNAs. Gene expression was measured using Step One Real-Time System (ABI) with SYBR Premix ExTaq. Relative gene expression levels were normalized against that of an internal reference gene (β actin) and calculated using the ΔΔCt method. Primers were designed by Primer-BLAST on the NCBI website. The expression of each gene was analyzed using three biological replications for each condition. Data were presented as the mean ± standard deviation (SD). SPSS 17.0 statistical software was used for statistical analysis of the data. The statistical significance of the differences between the groups was evaluated using student’s *t*-test. *p*-values of <0.05 were considered statistically significant (student’s *t*-test).

### 2.6. Prediction of miRNA Targets and Target Mimics

The 3749 miRNA mature sequences were collected from four rodent species in miRbase. We searched the conserved miRNAs with the same sequence in at least two species for the next analysis. Finally, a total of 1801 miRNA sequences were kept. The script GSTAr.pl could be used for the reverse complementary sequence of miRNA in other RNAs, and the minimum free energy (MEF) for miRNA-lncRNA/mRNA duplexes by RNAplex program [33,34,35]. We predicted the miRNA target and target mimics based on the published methods [36,37]. The following roles were used to obtain lncRNAs as miRNAs targets: no more than one mismatch or indel was allowed among the 9th and 12th positions from the 5′ end of miRNA sequences, the total number of mismatches or bulges in other regions was not allowed to exceed 4 nt, and no continuous mismatches were allowed. The roles for the miRNA target mimics were as follows: the number of mismatches or indels should be more than 1 and less than 6 from the 9th and 12th positions from the 5′ end of miRNA sequences, the perfect base pairing was required within 2nd and 8th positions of 5′ end of miRNA sequences, and the total mismatches and indels in other regions should be no more than 4. An in-house Perl script was used to implement the rules.

### 2.7. Construction of lncRNA-miRNA-mRNA Networks

The lncRNA-miRNA-mRNA networks were constructed based on the complementary pairs between miRNAs and lncRNAs and between miRNAs and mRNAs. The nodes in the networks consisted of miRNAs, lncRNAs acting as miRNA targets, lncRNAs acting as miRNA target mimics, mRNAs acting as miRNA targets, and mRNAs acting as miRNA target mimics. The lncRNA-miRNA-mRNA networks were visualized with Cytoscape 3.7.2 [38]. To assess the topological property, several measures were used. The node degree of a node *i* is the number of edges linked to *i*. The node betweenness is the number of shortest paths between pairs of nodes that run through node *i*. The average shortest path length is the average length of a shortest path between *n* and any other node. The closeness centrality is defined as the reciprocal of the average shortest path length.

### 2.8. Functional Prediction of DE-lncRNAs Responding to Hypoxia Based on the lncRNA-mRNA Co-Expression Networks

We constructed the co-expression network between mRNAs and lncRNAs using the published methods [39,40]. Briefly, the pipeline of constructing lncRNA-mRNA co-expression network was as follows: (1) The lncRNAs and mRNAs with their variances ranked in the top 75% of expression profiles were kept; (2) The *p*-values of Pearson’s correlation coefficient (*Pcc*) for each pair of genes were calculated by Fisher’s asymptotic test in the *WGCNA* library of R, and were adjusted by Bonferroni correction method; (3) The co-expression relationships with adjusted *p*-values (Fisher’s asymptotic test) of less than 0.05 and ranking in the top 5% and bottom 5% of *Pcc* were used for next analysis. The Bonferroni multiples test was executed using the multtest package in R. Cytoscape 3.7.2 was used for the visualization of the co-expression network [38].

Based on the co-expression networks between lncRNAs and mRNAs, we selected the mRNAs co-expressed with DE-lncRNAs to enrich their functions (GO terms and KEGG pathways) using the tools in website omicshare (https://www.omicshare.com/tools, latest accessed date: 1 November 2021).

## 3. Results

### 3.1. Global Identification of lncRNAs in E. fontanierii

**Figure 1:**
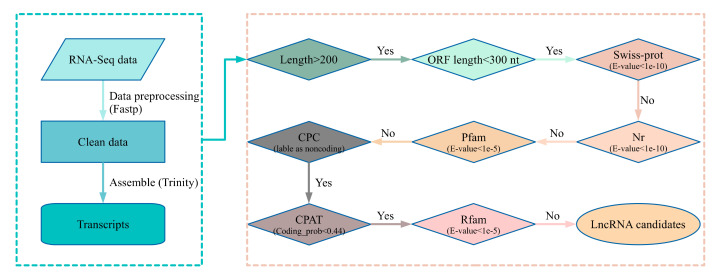
Pipeline for lncRNA identification. The left panel of the flow diagram showed the preprocessing and assemble of RNA-Seq data. The right panel displayed step-wise filters for transcripts from the left panel as input. In the first step of the right panel, the transcripts with a length of more than 200 nt were kept as input in the next step (Yes decision). For step two, the transcripts with the longest ORF (open reading frame) length less than 300 nt (100 aa) were used as input of the next step (“Yes” decision). In steps three, four, and give, the transcripts from the previous step that have matched items in the three protein-related databases under defined E-values were discarded (“No” decision), and the transcripts with no significant matches passed the three filters and were used as next step input. The transcripts labeled as noncoding by CPC were kept as input of the next step (“Yes” decision). The transcripts with a coding probability less than 0.44 calculated by CPAT were used for the next step input (“Yes” decision). The transcripts with matched items in Rfam (threshold E-value < 1e-5) were discarded (“No” decision), and the left transcripts were considered as lncRNA candidates. Swiss-prot: a high-quality annotated and non-redundant protein sequence database [42]. Nr: Non-redundant protein sequences database compiled by NCBI [41]. Pfam: the protein family database [27]. CPC: Coding Potential Calculator [29]. CPAT: Coding-Potential Assessment Tool [30]. Rfam: the RNA family database [31]. The tool Fastp (version: 0.20.1) was used for fastq preprocessing [23], Trinity was used for transcript assembly [24]. BLAST was used to search against databases, such as Swiss-prot, Nr, and Rfam [43].

The transcriptome data set were generated by RNA-Seq from liver and heart samples with three biological replicates for each condition. The total count of raw pair-end reads is 518.9 million. After removing the adapter-related and low-quality reads, 464 million pair-end clean reads were kept for the next analysis. The GC (guanine and cytosine) contents are about 49.73–52.48% and the reads satisfied with Q30 (99.9% base accuracy) are more than 85% across all samples (Table 1). All of the clean reads were assembled by Trinity, and 709,252 transcript contigs were generated [24]. The N50 and the average length of the transcript contigs were 1194 and 701.5 nt. By mapping clean reads into transcripts, the mapping ratios were 68.2–95.3% across samples. To identify mRNAs with functions, blastx searches against the Nr database were performed for all transcripts, and the BLAST output was used to annotate the functions of transcripts by Blast2GO. The transcripts with significant homologs in Nr database and GO terms were considered as mRNAs for our next analysis. To identify high confidence lncRNAs, all transcripts were subjected to a step-wise pipeline with a series of criteria (Figure 1). Briefly, the transcript with sequence length > 200 nt and the ORF length < 300 nt were kept for the next step. Several databases, such as Nr, Swiss-prot, Pfam, and Rfam, were used to exclude transcripts with homology with known proteins or ncRNAs (tRNA, rRNA, snRNA, and snoRNA) [27,31,41,42]. The software CPC (coding potential calculator) and CPAT for coding potential evaluation were used [29,30]. To minimize the background transcriptional noises, transcripts with low abundance (FPKM < 1) and expressed in only one sample were screened. Finally, 4877 lncRNA candidates satisfied with all criteria were identified.

### 3.2. Characteristics of E. fontanierii lncRNAs

**Figure 2:**
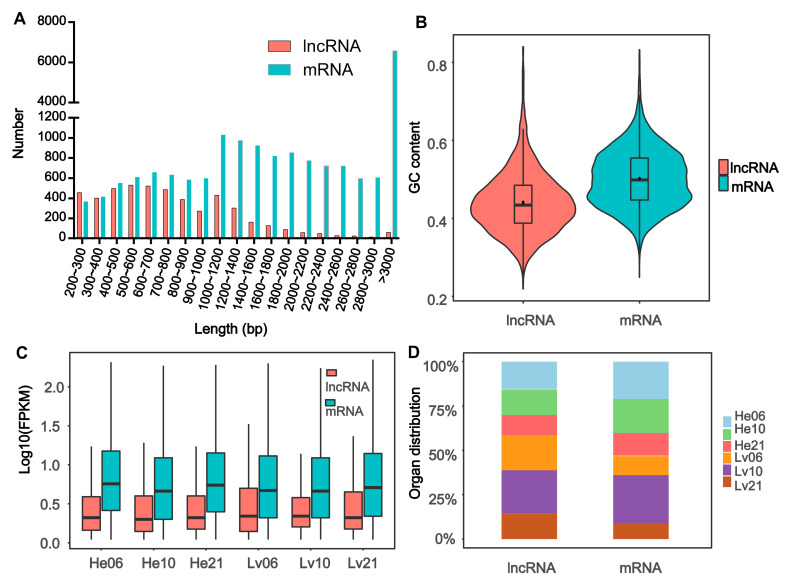
Characteristics of lncRNAs. (**A**) Length distribution of 4877 lncRNAs and 19,009 mRNAs. The *X*-axis represents the length and the *Y*-axis represents the number of lncRNAs and mRNAs with specific lengths. (**B**) The GC content distribution of lncRNAs and mRNAs. (**C**) The distribution of the expression levels for lncRNAs and mRNAs across different groups. (**D**) The organ distribution of lncRNAs and mRNAs with peak expression levels. LncRNAs: Long noncoding RNAs; mRNAs: messenger RNAs; GC content: the ratio of guanine plus cytosine, GC content percentage was calculated as Count (G + C)/Count (A + T + G + C) * 100%. FPKM: Fragments Per Kilobase of transcript per Million mapped reads; He06: heart tissue under 6.5% O_2_ concentration; He10: heart tissue under 10.5% O_2_ concentration; He21: heart tissue under 21% O_2_ concentration; Lv06: liver tissue under 6.5% O_2_ concentration; Lv10: liver tissue under 10.5% O_2_ concentration; Lv21: liver tissue under 21% O_2_ concentration.

The length of *E. fontanierii* lncRNAs ranged from 201 to 6053 nucleotides (nt), the majority of which were short (81.5%; 3973 out of 4877) in length (<1200 nt) (Figure 2A). The median and average length of lncRNAs were 706 and 849.8 nt, which is lower than that (2120 and 2701.6 nt) in mRNAs of *E. fontanierii*. It shows that lncRNAs have a shorter length than mRNAs, which is consistent with other species as expected [21]. The GC content of whole lncRNAs sequences was 43.44%, lower than the value (49.78%) observed in mRNAs (*p* < 2.2 × 10^−16^, Wilcoxon rank-sum test) (Figure 2B). The result is different from the lncRNA feature in naked mole-rat, in which the PCG (protein-coding genes) have lower GC content than that in lncRNAs [21]. This may be caused by the tissue- or species-specificity of lncRNAs. The expression levels of lncRNAs were also lower than that in mRNAs across tissues and treatments (Figure 2C), which is often observed in lncRNA researches. By comparing the numbers of lncRNAs with peak expression levels in the heart and liver tissues, we found that the number of lncRNAs with peak expression levels in the liver were larger than that in heart, while the numbers of mRNAs with peak expression levels were considerable in the two tissues (Figure 2D). We also found that both lncRNAs and mRNAs preferred to possess more peak expression levels in hypoxia (6.5% or 10.5% O_2_) than in normoxia (21% O_2_) in the two tissues.

### 3.3. Predication of Differentially Expressed lncRNAs

**Figure 3:**
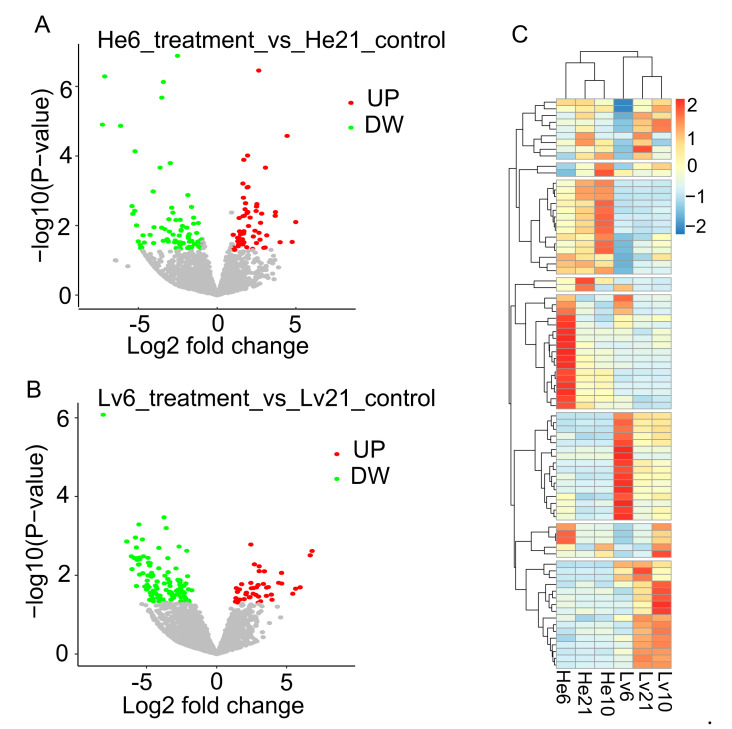
The volcano and heatmap plots of DE-lncRNAs. (**A**) Volcano plot for DE-lncRNAs under 6.5% vs. 21% O_2_ in heart. The *X*-axis shows the log2 fold changes of DE-lncRNAs, and *Y*-axis shows the −log10 (*p*-value) (Wald test). Red dots in the volcano plot represent the up-regulated DE-lncRNAs, and green dots represent the down-regulated DE-lncRNAs in 6.5% O_2_ treatment group compared with 21% O_2_ as control group in heart tissue. (**B**) Volcano plot for DE-lncRNAs under 6.5% O_2_ vs. 21% O_2_ in liver. (**C**) The heatmap of DE-lncRNAs, the expression levels were scaled by row. DE-lncRNAs: differential expressed lncRNAs; UP: up-regulated; DW: down-regulated. He6: heart tissue under 6.5% O_2_ concentration; He10: heart tissue under 10.5% O_2_ concentration; He21: heart tissue under 21% O_2_ concentration; Lv6: liver tissue under 6.5% O_2_ concentration; Lv10: liver tissue under 10.5% O_2_ concentration; Lv21: liver tissue under 21% O_2_ concentration.

To identify hypoxia-responsive lncRNAs of *E. fontanierii*, three paired comparisons among the three oxygen concentrations in two tissues were carried out: 10.5% O_2_ vs. 21% O_2_; 6.5% O_2_ vs. 21% O_2_; and 6.5% O_2_ vs. 10.5% O_2_. A total of 1128 lncRNAs were considered as differentially expressed lncRNAs (DE-lncRNAs) by DESeq2, which satisfied with a fold change > 2 and *p* < 0.05 [32]. For the comparisons between 6.5% O_2_ vs. 21% O_2_, 133 and 153 DE-lncRNAs were found in the heart and liver, separately (Figure 3A,B, Table 2), of which the number of downregulated lncRNAs (74, 110) were more than upregulated lncRNAs (59, 43). Most DE-lncRNAs were found when we compared the group of 6.5% O_2_ with the group of 10.5%O_2_, which showed more differences between the two hypoxic groups. 184 DE-lncRNAs were found shared among the same comparisons in two tissues or different upregulated/downregulated groups in the same tissues (Figure 3C). To validate the DE-lncRNAs, three DE-lncRNAs (TRNITY_DN164423_c1_g3, TRINITY_DN172918_c1_g1, and TRINITY_DN132528_c0_g1) were selected to be validated by quantitative real-time PCR (qRT-PCR) (Figure S1). The qRT-PCR results were generally consistent with RNA-seq data, in which TRNITY_DN164423_c1_g3 was significantly upregulated under chronic hypoxia compared with normoxia, TRINITY_DN172918_c1_g1 was upregulated in hypoxia compared with normoxia, and TRINITY_DN132528_c0_g1 was upregulated in acute hypoxia compared with chronic hypoxia and normoxia (*p* < 0.05, student’s *t*-test).

### 3.4. Prediction of lncRNAs as Potential Targets or Target Mimics of miRNAs

**Figure 4:**
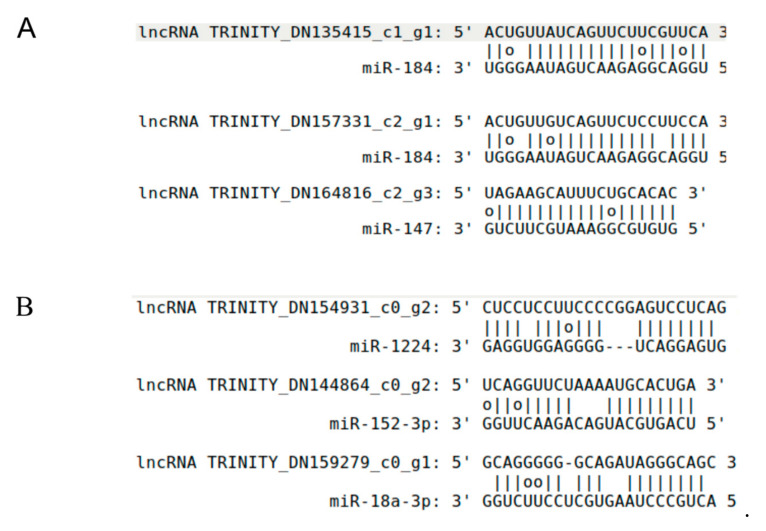
The predicted alignments between miRNAs and lncRNAs. (**A**) LncRNAs predicted as putative miRNA targets. The rules of miRNA targets were showed as follows: at most, one mismatch or indel was allowed between the 9th and 12th positions of the 5′ end of miRNA sequence, the total number of bulges or mismatches in the other regions was not allowed to exceed 4 nt, and no continuous mismatches were allowed. In this figure, miR-184 has perfect matches with the three lncRNA targets between 9th and 12th from miRNA 5′ end, and has 0~2 discontinuous mismatches in other regions. G-U pairs were not considered as a mismatch for RNA molecular. (**B**) LncRNAs are predicted as putative miRNA target mimics. The rules of miRNA target mimics were showed as follows: the number of mismatches or indels should be larger than 1 and less than 6 between 9th and 12th positions of the 5′ end of miRNA sequence, perfect nucleotide pairing was required between the 2nd and 8th positions of the 5′ end of miRNA sequences, and the number of mismatches and indels should be no more than 4 nt in other regions. For the miRNA miR-1224, miR-152-3p and miR-18a-3p, all of them have 2~3 mismatches or indels between 9th and 12th from miRNA 5′ end, perfect pairing between the 2nd and 8th positions of the 5′ end of miRNA, and 2~3 nt mismatches and indels in other regions.

Studies showed that lncRNAs can modulate gene expression by playing roles as miRNA targets or target mimics [17,18]. To investigate the lncRNA roles in the miRNA regulation network, we predict the potential target or target mimics in the lncRNA set applying a computational pipeline [36,37]. For potential lncRNAs as miRNA targets, 4922 interactions were found between 666 miRNAs and 2151 lncRNAs. For example, lncRNA TRINITY_DN135415_c1_g1 and TRINITY_DN157331_c2_g1 acted as targets of miR-184, and both of them had perfect pairing in middle regions (from 9th to 12th) of miRNA (Figure 4A). There were 639 lncRNA-miRNA interactions, in which 439 lncRNAs were predicted as target mimics of 319 miRNAs. For example, miRNA-1224, miR-152-3p, and miR-18a-3p had bulges in positions between 9th and 12th of miRNA with the alignments of target mimics of lncRNA TRINITY_DN154931_c0_g2, TRINITY_DN144864_c0_g2, and TRINITY_DN159279_c0_g1, respectively (Figure 4B). For the DE-lncRNAs as miRNA target mimics, 137 interactions were predicted between 119 miRNAs and 92 lncRNAs. Five miRNAs, such as miR-203, miR-326, miR-18, miR-344, and miR-185 possessed three DE-lncRNA target mimics. For the DE-lincRNAs as miRNA target, 1102 interactions between 405 miRNAs and 471 lncRNAs were found, in which miR-574, miR-760, and miR-328 have more than 20 DE-lncRNA targets for each of them.

### 3.5. Construction of lncRNA-miRNA-mRNA Networks

**Figure 5:**
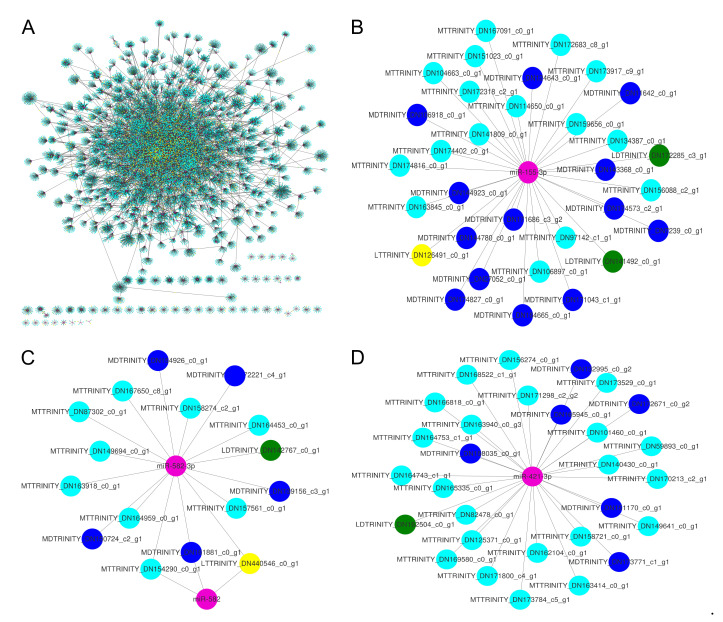
The transcriptome-wide miRNA-regulated networks. pink nodes, miRNAs; yellow nodes, lncRNAs that may be miRNA targets; green nodes, lncRNAs that may be miRNA target mimics; cyan nodes, mRNAs that may be miRNA targets; blue nodes, mRNAs that may be miRNA target mimics; grey edges, correlations. The labels of nodes were prefixed with specific abbreviations, such as MT for mRNA target, LT for lncRNA target, MD for mRNA mimics, and LD for lncRNA mimics. (**B**–**D**) were extracted from (**A**).

Research has shown that miRNAs participate in complex networks including their targets and target mimics, and lncRNAs could act as targets or target mimics of miRNAs [18,44,45]. To infer the functions of lncRNAs acting as targets or target mimics of miRNAs, we constructed the networks of lncRNA-miRNA-mRNA, in which miRNAs connected both mRNAs and lncRNAs simultaneously. The networks had 18,289 nodes connected by 104,422 edges, in which the nodes included 2282 lncRNAs (acting as miRNA targets or target mimics), 688 miRNAs, and 15,319 mRNAs (acting as miRNA targets or target mimics) (Figure 5A). There were 638 interactions between 318 miRNAs and 438 lncRNAs acting as miRNA target mimics and 4911 interactions between 665 miRNAs and 2141 lncRNAs acting as miRNA targets. In addition, it was found that 6027 mRNAs acted as 617 miRNA target mimics, and 14,888 mRNAs acted as 688 miRNA targets in the networks. To display the node details in-network, several sub-networks from the whole networks were extracted as examples. In Figure 5B–D, miR-155-3p, miR-582-3p, and miR-432-3p nodes were connected with four types of RNAs, including lncRNAs as miRNA targets/target mimics, mRNA as miRNA targets/target mimics. 673 miRNA, 16 lncRNAs acting as miRNA targets, 2828 mRNAs acting as miRNA targets, and 5 mRNAs acting as miRNA target mimics were identified as hub genes (each hub gene has at least ten other RNAs as partners). The topological features, ‘degree’, ‘node betweenness’, and ‘closeness centrality’, and ‘average shortest path length’ were used to profile the topological nodes. The scope of Average shortest path length for mRNA, lncRNA, and miRNAs ranged from 2.79 to 5.75, and most miRNAs had a lower value of average shortest path length than other RNA types (Figure S2A), suggesting that miRNAs were connected with other nodes with closer path length. By analyzing the other three topological features, we found that miRNAs and other RNA types exhibit different density peaks and that the density peaks of mRNAs lag behind other RNA types (Figure S2B–D), suggesting that miRNAs possess higher node betweenness, closeness centrality, and degree than other RNA types (Kolmogorov-Smirnov test, *p* < 2.2 × 10^−16^). Closeness centrality is a measure of how fast information spreads from a given node to other reachable nodes in the network, suggesting miRNAs regulated other RNAs with shorter path length.

**Figure 6:**
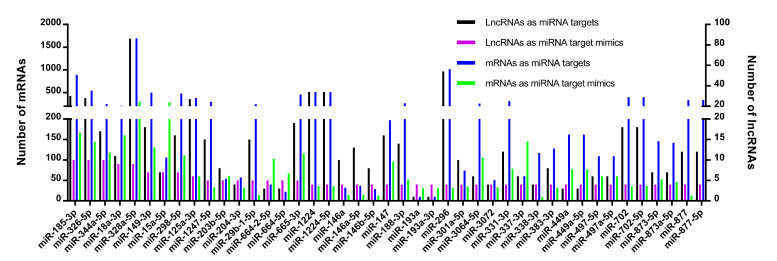
The number of alignments formed by miRNA-DE-lncRNA and miRNA-mRNA duplexes. The *X*-axis legend shows the miRNAs. The *Y*-axis legend represents the number of lncRNAs (**right**) or mRNAs (**left**) that function as miRNA target or target mimics. The different colors of bars indicate different types of RNAs.

To search the patterns of DE-lncRNA-miRNA-mRNA networks, we compared the number of DE-lncRNAs as miRNA targets/target mimics, mRNAs as miRNA targets/target mimics, in which the mRNAs were connected to DE-lncRNAs indirectly intermediated by miRNAs. The number of those four RNA types was distributed unevenly for every miRNA. The number of DE-lncRNAs as miRNA targets was more than the number of DE-lncRNAs as miRNA target mimics for a major of miRNAs (Figure 6). For a small part of miRNAs, the DE-lncRNAs as miRNA target mimics had a larger number than the DE-lncRNAs as miRNA targets, such as miR-193, miR-664-5p, and miR-204-3p.

**Figure 7:**
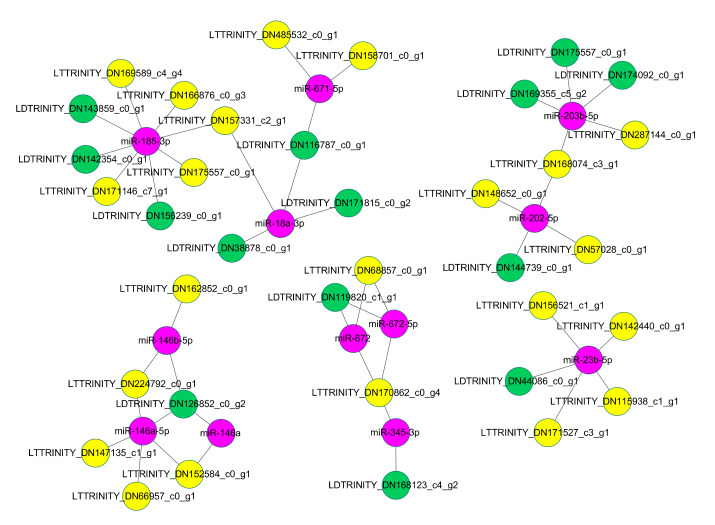
Representative regulatory networks of miRNA-lncRNA duplexes. Pink nodes, miRNAs; yellow nodes, lncRNAs that may be miRNA targets; green nodes, lncRNAs that may be miRNA target mimics. The labels of nodes were prefixed with specific abbreviations, such as MT for mRNA target, LT for lncRNA target, MD for mRNA mimics, and LD for lncRNA mimics. Grey edges: correlations.

In addition, some miRNAs could bind multiple DE-lncRNAs (Figure 7). For example, lncRNA TRINITY_DN485532_c0_g1 and TRINITY_DN158701_c0_g1 acted as the target of miR-671-5p, while TRINITY_DN116787_c0_g1 acted as the target mimic of the miRNA. For some DE-lncRNAs, they could be bound by multiple miRNAs. For example, lncRNA TRINITY_DN157331_c2_g1 could be bound by miR-185-3p and miR-18a-3p, and lncRNA TRINITY_DN170862_c0_g4 could be bound by miR-672, miR-672-5p, and miR-345-3p. Some DE-lncRNAs could act as target and target mimics of different miRNAs at the same time. For example, TRINITY_DN162199_c1_g2 could act as the target of miR-326, miR-330-5p, and miR-296-3p, and this lncRNA could also act as the target mimics of miR-328a and miR-147.

### 3.6. Potential Functions of lncRNAs Based on Co-Expression Networks

To explore the functions of lncRNAs responding to hypoxia in liver and heart tissues of *E. fontanierii*, we firstly constructed the co-expression networks of lncRNA-mRNA, and lncRNAs’ functions were assigned by enriching the functions of the mRNAs that were associated with lncRNAs. There were 8893 nodes in co-expression networks, including 917 lncRNA nodes and 7976 mRNA nodes. There were 5650 edges (5554 positive correlations and 96 negative correlations) within 749 lncRNA nodes, 80,577 edges (70,064 positive correlations and 10,513 negative correlations) connecting lncRNAs (number: 895) and mRNAs (number: 5960), and 651,565 edges (497,753 positive correlations and 153,812 negative correlations) linking mRNAs (number: 7922).

**Figure 8:**
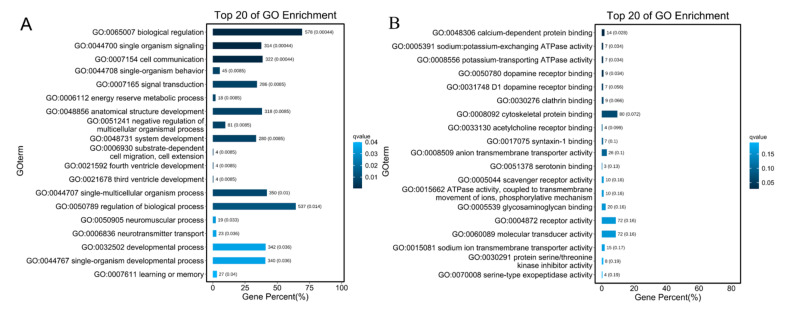
The top 20 of GO enrichment of DE-lncRNAs co-expressed with mRNAs in heart. (**A**) The top 20 of GO enrichment for Biological Process. (**B**) The top of GO enrichment for molecular function. The numbers to the right of bars mean the number of DE-lncRNAs in specific enriched GO terms and the numbers in brackets mean the *q*-value.

By enriching the GO terms for mRNAs that were co-expressed with DE-lncRNAs found in the heart, the main GO terms were biological regulation, metabolic process, regulation of biological process, response to stimulus, catalytic activity, molecular function regulator. We found the most enriched GO terms were associated with biological regulation (578 genes) energy reserve metabolic process (18 genes), negative regulation of multicellular organismal process, system development (280 genes), cell communication (322 genes), calcium-dependent protein binding (14 genes) (Figure 8A,B). The main enriched KEGG pathways were protein digestion and absorption (14 genes), microRNAs in cancer (24 genes), insulin signaling pathway (24 genes) (Figure S3). In total, 23 genes co-expressed with DE-lncRNAs were annotated by the GO term “response to hypoxia”, which may play key roles in hypoxia adaption in subterranean animals and may be regulated by DE-lncRNAs.

By enriching GO terms of mRNAs that were co-expressed with DE-lncRNAs in the liver, we found the most enriched GO terms were oxoacid metabolic process (115 genes), lipid metabolic/biosynthetic process (119/71 genes), catalytic activity (406 genes), cofactor binding (46 genes), endoplasmic reticulum (139 genes), mitochondrion (136 genes) (Figure S4A–C). The most enriched pathways were fatty acid metabolism (19 genes), steroid biosynthesis (10 genes), drug metabolism/other enzymes (24 genes), citrate cycle (TCA cycle) (11 genes) (Figure S4D). The GO term “acute-phase response” was enriched, and it was assigned to 15 genes co-expressed with DE-lncRNAs, suggesting that DE-lncRNAs may play roles in acute hypoxia response.

## 4. Discussion

In this article, we predicated the lncRNAs in *E. fontanierii* in two tissues heart and liver. Thousands of lncRNAs were found in RNA-Seq data by stringent pipelines. Multiple filters were used to remove the coding potential transcripts. Partial transcripts were removed by sequence features, such as long open reading frames and Pfam motifs, which could screen coding-potential sequences with no homologous in protein databases. Alignment tool Blastx was used to search known protein homologous of our transcripts against two protein databases, Nr and Swiss-Prot. In addition, two machine learning tools (CPC and CPAT) were used to filter potential coding transcripts based on sequence intrinsic features [29,30]. Although our pipelines were designed from different aspects to identify reliable lncRNAs, some lncRNAs containing short ORFs maybe not be screened by our filters, which may code small peptides. Evidence shows that there exists a small peptide world in lncRNAs [46,47,48,49]. It is worthful to explore the phenomenon of lncRNAs coding short peptides.

By analyzing the characters of lncRNAs, we found that *E. fontanierii* lncRNAs often have a shorter length, lower GC contents, and expression levels than that of mRNAs, which were consistent with other species studies of lncRNAs [50]. The different pattern of GC contents for lncRNAs and mRNAs between *E. fontanierii* and naked mole-rat may be caused by the species specificity, and lncRNAs often have poor conservation in sequences among different species, which may be one reason for inconsistency sequence features. By comparing the peak expression levels of lncRNAs across groups, we found that lncRNAs with peak expression levels under hypoxic conditions have a larger number than that of normoxic conditions, and a larger number of lncRNAs with peaks expressed were found in the liver than in the heart. It showed that lncRNAs prefer to possess higher expression levels under hypoxia in the liver, which may be caused by the tissue-specific expression of lncRNAs. The liver could detoxify various metabolites, synthesize protein, and produces biochemicals necessary for digestion [51]. The response to hypoxia of liver may change the normal metabolic process, and more lncRNAs may be needed to regulate gene expression.

As lncRNAs could regulate the gene expressions in different ways, studies showed that lncRNAs could be miRNA targets or target mimics [18,52,53]. For example, lncRNA-TINCR acts as a competitive endogenous RNA by sponging miR-761 in the migration of mesenchymal stem cells [52]. Here, we explore those lncRNAs in *E. fontanierii* acting as the targets or target mimics of miRNAs by computational methods. For example, HIF1A was predicted as the target of miRNA miR-154-5p, miR-129b-5p, miR-1912-3p, and miR-1188-5p, while 12 DE-lncRNAs also had duplexes with those miRNAs (Figure S5), suggesting that the same miRNAs may regulate HIF1A and DE-lncRNAs under hypoxia. We found that some lncRNAs with the mismatches or bulges in the 9th to 12th positions of miRNA-lncRNA pairing sites could not be cleaved by the miRNA-associated silencing complex. By bioinformatic methods, 439 lncRNAs were predicted as target mimics of 319 miRNAs in *E. fontanierii*. Our results showed that the lncRNAs acting as miRNA target mimics (sponges or decoys) were popular in *E. fontanierii*, which could act as the regulators of miRNAs. Based on ceRNA (competing endogenous RNA) hypothesis, lncRNAs that act as target mimics of miRNAs sequester miRNAs and favor the repressed mRNA targets. In our lncRNA-miRNA-mRNAs networks, 438 lncRNAs act as target mimics of 318 miRNA and the 318 miRNAs have 12,982 mRNA targets. Those lncRNAs could regulate mRNA functions with ceRNA manners. We also found that some DE-lncRNAs could work as target mimics of miRNA. It suggests that DE-lncRNAs responding to hypoxia may regulate mRNA functions by ceRNA mechanism. In the lncRNA-miRNA-mRNA networks, one miRNA may be targeted by different mRNAs and lncRNAs, and one mRNA or lncRNA may target multiple miRNAs, which extended the complexity of gene regulation networks.

To search the function of lncRNAs responding to hypoxia, we constructed co-expression networks of lncRNA-mRNA and enriched the functions of mRNAs with co-expressed DE-lncRNAs. The enriched GO terms and KEGG pathways were assigned to those corresponding lncRNAs. We found that the most enriched GO term biological regulation was found for the DE-lncRNA in the heart, suggesting that many lncRNAs may participate in the biological regulation under hypoxic stress. Biological regulation is what allows an organism to handle the effects of a perturbation, modulating its own constitutive dynamics in response to particular changes in internal and external conditions [54]. The GO term biological regulation was also enriched in naked mole-rat in response to hypoxia [55]. The DE-lncRNAs involved in biological regulation may maintain homeostasis to avoid disturbances in metabolic process under hypoxic conditions. Other GO terms, such as energy reserve metabolic process, anatomical structure development, system development, and three/fourth ventricle development were associated with heart functions, suggesting that lncRNAs in the heart may play important roles in heart development under hypoxia. In another study in mole rat *Spalax,* energy-saving response is found as a key adaptation to low oxygen levels [56]. In our study, many energy-related GO terms and KEGG paths were enriched, such as energy reserve metabolic process, insulin resistance, citrate cycle (TCA cycle), glucagon signaling pathway, and pyruvate metabolism. It suggested that DE-lncRNAs have functions in regulating energy processes, which maybe contribute to the adaption to hypoxia. When the mRNA co-expressed with DE-lncRNAs were enriched, many metabolic processes were found associating with liver functions, such as small molecule metabolic process, lipid metabolic process, fatty acid biosynthetic process, and sterol biosynthetic process. Lipid metabolism is modified during hypoxia or in tumor cell, and study in *Spalax* shows that genes responding to hypoxia are significantly associated with lipid metabolism [57,58]. It suggested that DE-lncRNAs in the liver may act relevant roles in liver main functions under hypoxia when those DE-lncRNAs were co-expressed with mRNAs for metabolic and biosynthetic processes.

Our exploration in lncRNA-miRNA-mRNA networks showed the potential regulation patterns of lncRNAs. We found that some DE-lncRNAs acted as the target or target mimics of miRNAs that were associated with hypoxic stress in published studies, such as, miR-185-3p, miR-671, miR-18, miR-664, and miR-15 [59,60,61,62,63]. For example, the inhibition of miR-15 protects against cardiac ischemic injury [63]. DE-lncRNA TRINITY_DN162625_c0_g1 may repress the function of miR-15 by acting as miR-15 target mimic. The endogenous reduction of miR-185 accelerates cardiac function recovery in mice [64]. DE-lncRNA TRINITY_DN156239_c0_g1, TRINITY_DN143859_c0_g1, and TRINITY_DN142354_c0_g1 were predicted as the target mimics of miR-185, which may repress the function of miR-185 to help the cardiac function stability under hypoxia. Those miRNAs play roles in hypoxia-associated diseases or cancer, suggesting that DE-lncRNAs could regulate those miRNAs based on competing for endogenous RNA (ceRNA)-mediated regulatory mechanisms, and further affect the functions of mRNA target. Our results provide a comprehensive view of miRNA-regulated networks and indicate that lncRNAs can participate in the regulatory interactions as miRNA target or target mimics. Our study helps to understand the complexity of hypoxia adaption, forms the basis of further studies of hypoxia adaptation in *E. fontanierii*, and has potential biomedical applications. Our methods in lncRNA identification and miRNA target prediction could be used by other species to extend the knowledge about lncRNAs and their functions in miRNA regulation network.

## Figures and Tables

**Table 1:** Mapping Statistics of Clean Reads with Assembled Transcripts.

Samples ^a^	Clean Read	Clean Read Ratio (%)	G + C (%)	≥Q30 (%)	Mapped Reads	Mapped Ratio (%) ^b^
**Liver** **Normoxia** **(21% O_2_)**	27,854,142	93.94	51.32	85.01	19,951,468	71.63
	26,718,742	93.29	52.06	85.16	19,498,631	72.98
	29,040,231	92.45	51.75	85.1	21,118,333	72.72
**Liver** **Chronic Hypoxia** **(10.5% O_2_)**	27,103,408	86.76	52.48	85.17	18,487,988	68.21
	27,384,840	81.35	51.99	85.12	19,445,390	71.01
	24,116,984	99.75	50.47	92.92	18,714,000	77.60
**Liver** **Acute Hypoxia** **(6.5% O_2_)**	23,280,246	90.95	52.19	85.02	17,227,546	74.00
	25,090,462	99.46	50.65	93.3	19,308,428	76.96
	28,633,359	99.46	49.73	93.3	21,722,527	75.86
**Heart** **Normoxia** **(21% O_2_)**	24,686,829	92.59	51.88	85.01	18,658,425	75.58
	25,557,787	95.05	51.98	85.03	19,446,190	76.09
	24,965,067	91.58	50.57	85.15	18,245,211	73.08
**Heart** **Chronic Hypoxia** **(10.5% O_2_)**	20,584,903	80.19	50.39	85.05	15,443,520	75.02
	20,638,228	82.54	50.48	85.88	15,654,399	75.85
	39,391,311	88.58	50.49	85.2	28,284,097	71.80
**Heart** **Acute Hypoxia** **(6.5% O_2_)**	22,190,427	81.75	50.89	85.17	16,216,024	73.08
	20,402,708	80.68	51.23	85.45	19,445,390	95.31
	26,388,845	82.04	50.79	85.25	19,385,947	73.46

Notes. **^a^** 21% O_2_, 10.5% O_2,_ and 6.5% O_2_ include three biological replicates of *E. fontanierii* for the three oxygen concentrations; **^b^** the mapped ratio represents the ratio of mapped reads to clean reads.

**Table 2:** DE-lncRNA Number.

DE-lncRNAs Sets (%)	All DelncRNAs	Upregulated	Downregulated
Heart 6.5 versus 21	133	59	74
Heart 6.5 versus 10.5	278	167	111
Heart 10.5 versus 21	100	19	81
Liver 6.5 versus 21	153	43	110
Liver 6.5 versus 10.5	465	165	300
Liver 10.5 versus 21	328	34	294

## Data Availability

Not applicable.

## Supplementary Materials

The following are available online at https://www.mdpi.com/article/10.3390/cimb43030132/s1, Figure S1: RT-qPCR validations for DE-lncRNAs. (A) lncRNA TRINITY_DN13528_c0_g1. (B) lncRNA TRINITY_DN164423_c1_g3. (C) lncRNA TRINITY_DN172918_c1_g1. Asterisks above bars indicate a significant difference in the gene expression among samples (* *p*-value < 0.05, student’s *t*-test); Figure S2: The distribution of topological properties. (A) The density distribution of average shortest path length for different RNA types. (B) The density distribution of node betweenness for different RNA types. (C) The density distribution of closeness centrality for different RNA types. (D) The density distribution of degree for different RNA types. The lncRNA-miRNA-mRNA network were analyzed by NetwrokAnalyzer from the Tools of Cytoscape 3.7.2 to obtain the topological properties; Figure S3: The enriched pathways of DE-lncRNAs co-expressed with mRNAs in heart. The numbers to the right of bars mean the number of DE-lncRNAs in specific enriched pathway and the numbers in brackets means the q-value; Figure S4: The top 20 of GO/KEGG enrichment of DE-lncRNAs co-expressed with mRNAs in liver. (A) The top 20 of GO enrichment for Biological Process. (B) The top 20 of GO enrichment for molecular function. (C) The top 20 of GO enrichment for cellular component. (D) The top 20 of KEGG path enrichment. The numbers to the right of bars mean the number of DE-lncRNAs in specific enriched GO/pathway and the numbers in brackets means the q-value; Figure S5: Regulatory networks of HIF1A-miRNA-lncRNA duplexes. Pink nodes: miRNAs. Yellow nodes: lncRNAs that may be miRNA targets. Cyan nodes: mRNAs (HIF1A) that may be miRNA targets. Grey edges: correlations.

## Abbreviations

Q30	99.9% base accuracy
DE-lncRNAs	Differentially Expressed long noncoding RNAs
Lv6	liver tissue under 6.5% O_2_ concentration
Lv10	liver tissue under 10.5% O_2_ concentration
Lv21	liver tissue under 21% O_2_ concentration
He6	heart tissue under 6.5% O_2_ concentration
He10	heart tissue under 10.5% O_2_ concentration
He21	heart tissue under 21% O_2_ concentration
KEGG	Kyoto Encyclopedia of Genes and Genomes
Pfam	Pfam protein domain database
Nr	RefSeq Non-redundant protein sequences
GO	Gene Ontology.
FPKM	Fragments Per Kilobase of transcript per Million mapped reads
