# Supplementary figures and images for: Identification and Functional Analysis of lncRNAs Responsive to Hypoxia in Eospalax fontanierii
Source: Curr Issues Mol Biol. 2021 Nov 5;43(3):1889–905. doi: 10.3390/cimb43030132 (PMC8929107; doi:10.3390/cimb43030132)

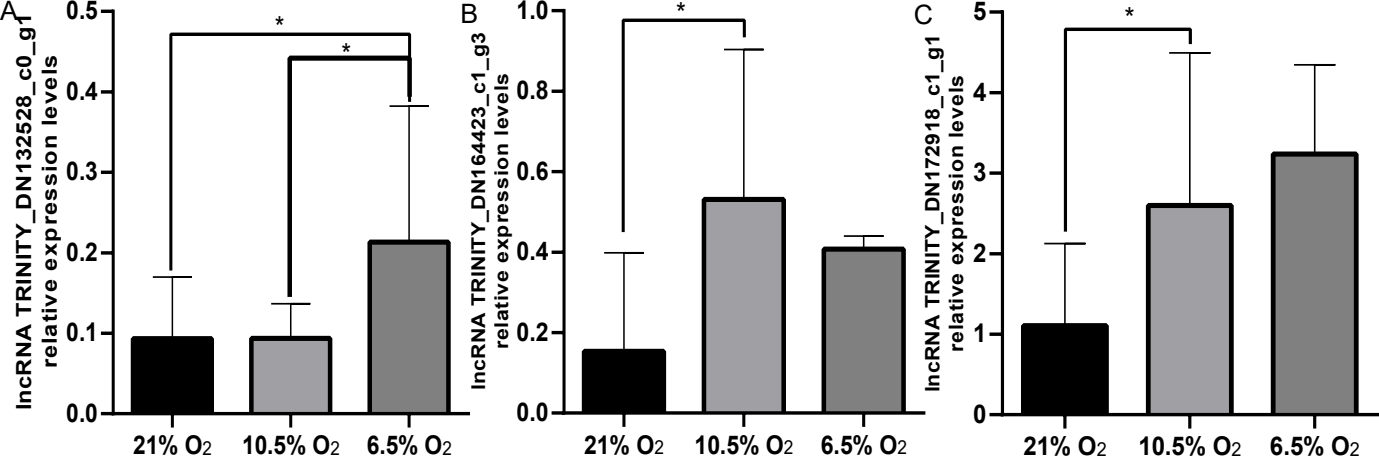

Supplement: Supplementary file 1 [file cimb-43-00132-s001.zip › FigureS1_qprc.pdf]

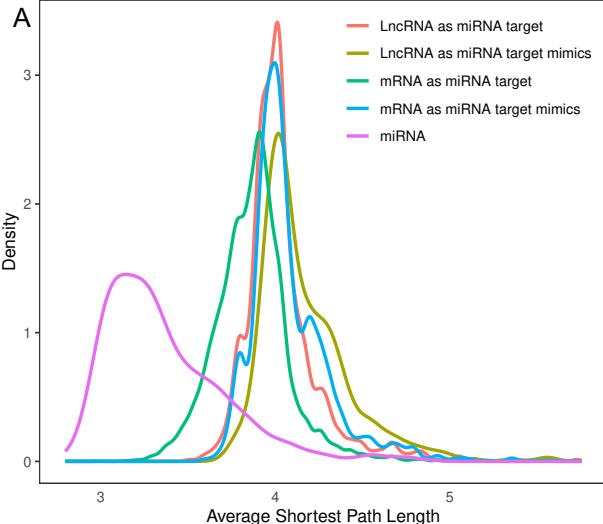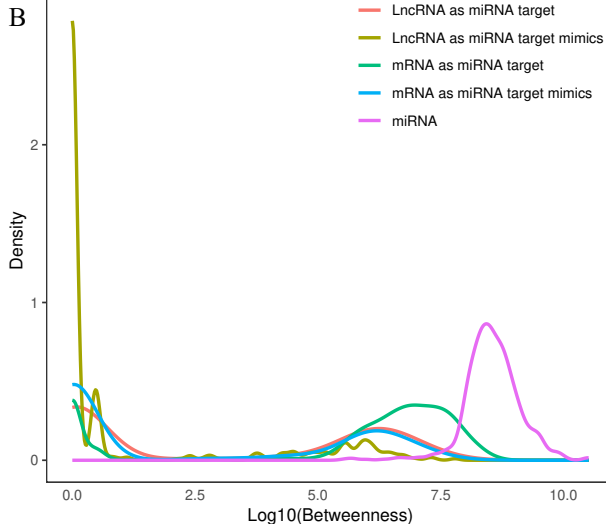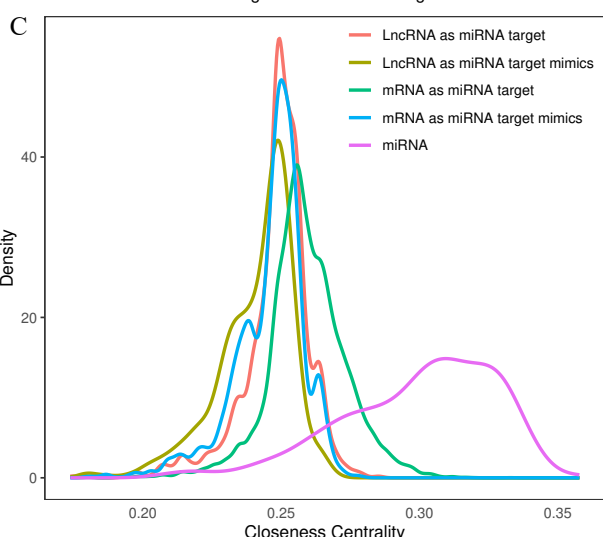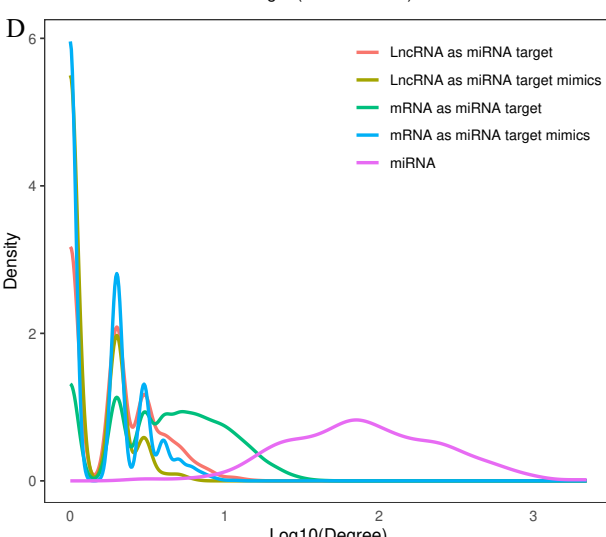

Supplement: Supplementary file 1 [file cimb-43-00132-s001.zip › FigureS2_densityHist.pdf]

# Top 20 of KEGG Enrichment

Pathway

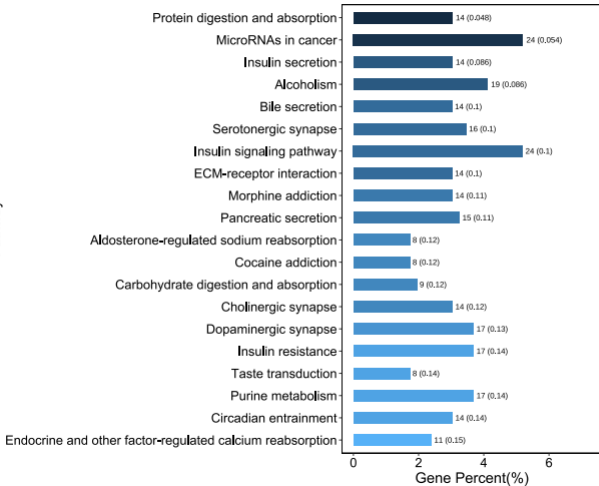

Supplement: Supplementary file 1 [file cimb-43-00132-s001.zip › FigureS3_.pdf]

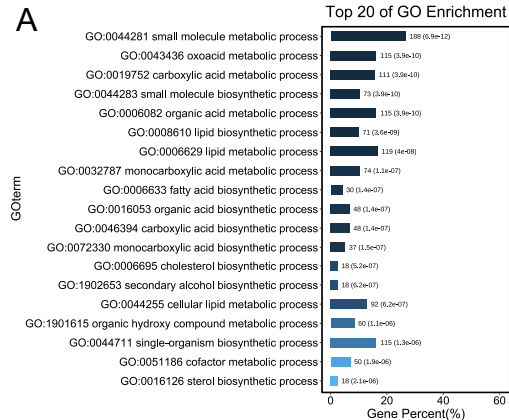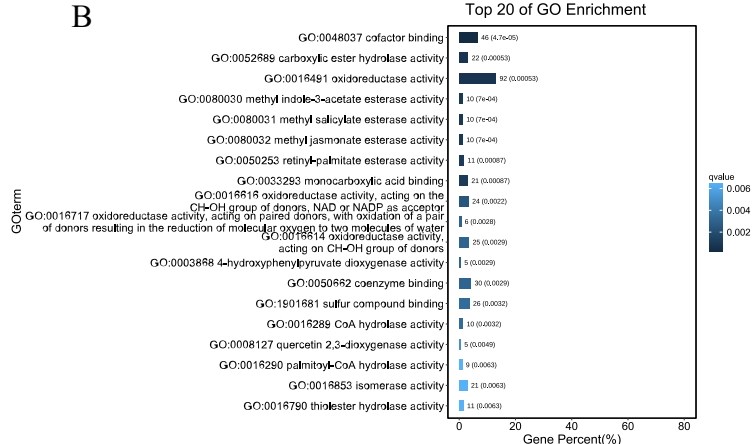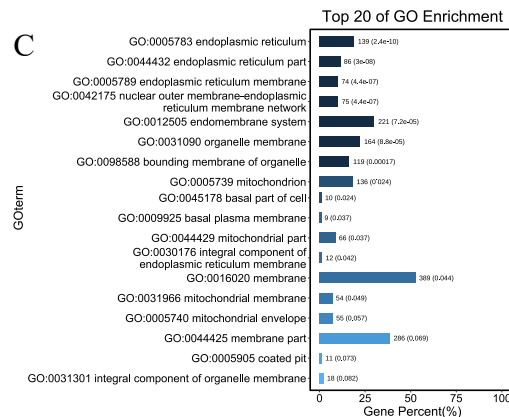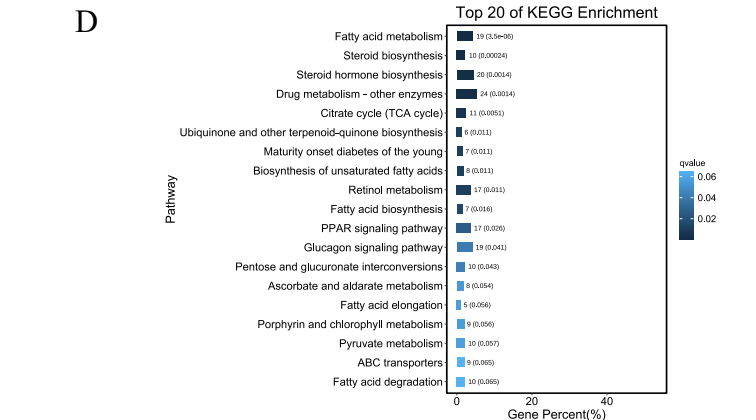

Supplement: Supplementary file 1 [file cimb-43-00132-s001.zip › FigureS4_V2.pdf]

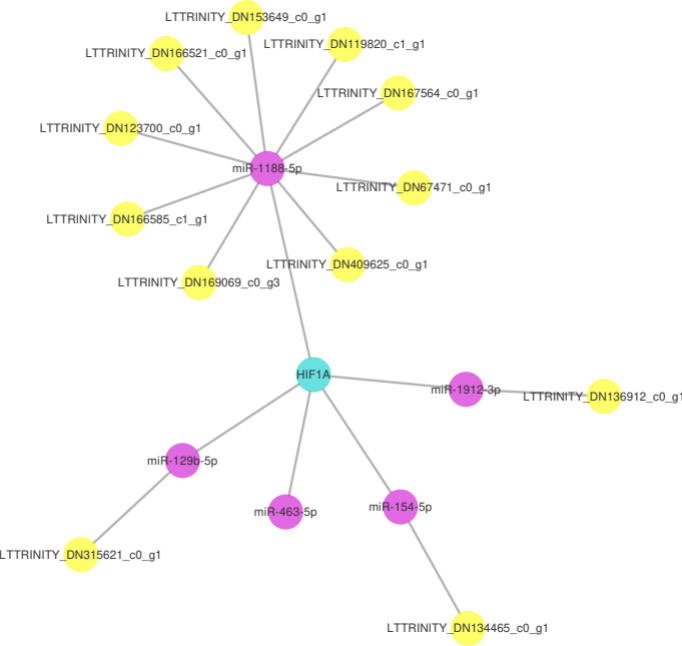

Supplement: Supplementary file 1 [file cimb-43-00132-s001.zip › FigureS5_HIF_network.pdf]
